# Efficient simultaneous adsorption-biodegradation of high-concentrated N,N-dimethylformamide from water by *Paracoccus denitrificans-*graphene oxide microcomposites

**DOI:** 10.1038/srep20003

**Published:** 2016-02-01

**Authors:** Yuan Zheng, Dongyun Chen, Najun Li, Qingfeng Xu, Hua Li, Jinghui He, Jianmei Lu

**Affiliations:** 1 College of Chemistry, Chemical Engineering and Materials Science, Collaborative Innovation Center of Suzhou Nano Science and Technology, Soochow University, Suzhou, 215123, China

## Abstract

Water contamination becomes one of the most pervasive environmental issues all over the world in recent years. In this paper, the functionalization of graphene oxide (GO) with copolymers containing methacrylic acid (MAA) and butyl methacrylate (BMA) was investigated to prepare a new microcomposite material (PGO) *via* free radical solution polymerization. PGO was used for the adsorption of N,N-dimethylformamide (DMF) from aqueous solution by utilizing the characteristics of ultralarge surface and the Van der Waals force between DMF molecules and polymers on the surface of PGO. Besides, PGO was used not only a high-capable adsorbent but also a carrier for the immobilization of *Paracoccus denitrificans* cells in the treatment of high-concentrated DMF. Bacterial cells could immobilized on the PGO (PGO@*P. denitrificans*) stably by covalent coupling process after acclimatization and high-concentrated DMF (2000 mg/L) could be removed completely and relatively rapidly from aqueous solutions by the simultaneous adsorption-biodegradation (SAB) process of PGO@*P. denitrificans*. Furthermore, the excellent recycle performance of PGO@*P. denitrificans* made the whole process more economical and practical.

As a versatile organic solvent, N,N-dimethylformamide (DMF) is widely used in pharmaceutical, chemical and textile industries due to its excellent miscibility with both water and most organic solvents^1^. Hence, high concentrations and considerable amounts of DMF are commonly found in many industrial effluents even after its recovery, which cause adverse toxic effects on both environment and human health^2^,^3^,^4^. Therefore, many methods have been studied in the past few years to deal with DMF in wastewater, such as distillation, adsorption, biodegradation, chemical oxidation and solvent extraction^5^. Biodegradation has considered to be the most practical, economical, environmentally friendly and promising approach^6^,^7^,^8^,^9^,^10^,^11^. Moreover, numerous researches based on the biodegradation of different organic pollutants by using immobilization technique have been reported^12^,^13^,^14^. The biodegradation process combining with immobilization technique owns more advantages than free cells, including enhancing the stability of microbial cells, avoiding the biomass-liquid separation and discontinuous process operation. However, the high-concentrated DMF wastewater may have toxic to bacterial cells and the removal efficiency of the DMF was improved hardly.

Recently, simultaneous adsorption and biodegradation (SAB, immobilize on adsorbents) was common used in the treatment process of wastewater containing high-concentrated DMF. Therefore, adsorbents play an important role in the performance of adsorption process. Some traditional adsorbents are popular in removal of organic contaminants in water treatment^15^. However, these adsorbents have many disadvantages, such as high cost and low reusability. Therefore, a considerable amount of researches have focused on various commercially available and regenerated adsorbents instead of traditional adsorbents^16^. In addition to inorganic absorption materials, some nanomaterials are also popular in environmental remediation, which provided a lower cost, greater sensitivity, smaller sizes, higher efficiency than traditional adsorbents^17^.

As a fascinating new nanomaterial, graphene oxide (GO) which is an amazing derivative of graphene, has attracted great attention all over the world recently^18^,^19^,^20^,^21^,^22^,^23^,^24^. In addition, GO has been used as an effective adsorbent on the treatment of different kinds of organic wastewater owing to its extraordinary mechanical strength and relatively large specific area^25^,^26^,^27^,^28^,^29^. And because GO is heavily decorated by oxygen-containing groups (carboxyl, hydroxyl, epoxy) on their basal planes and edges, various of polymers can be modified on the surface of GO easily, which cause a higher efficiency to the adsorption of organic contaminants than GO^30^,^31^,^32^,^33^,^34^,^35^. However, as a normal adsorbent, GO can reach a maximal equilibrium adsorption amount of DMF but cannot remove DMF completely.

Here, in order to resolve the problems mentioned above, the fabrication of the new adsorbent microcomposite material (PGO) for the efficient removal of DMF from water is reported. The key strategy is to use copolymers containing methacrylic acid (MAA) and butyl methacrylate (BMA) to modify the surface of GO, leading to the formation of GO@poly(MAA-*co*-BMA) (PGO) consisting abundant oxygen-containing groups (Fig. 1). In addition, the unique PGO also showed a favorable adsorption property for its ultra large surface and Van der Waals force between DMF molecules and polymers on its surface, which was employed to be the superior carrier of *P. denitrificans* (ATCC 19367) (had already been reported on the biodegradation of DMF^9^) for its large loading amount of bacterial cells. The immobilization process was achieved by covalent coupling process^36^, which made the bacterial cells immobilized on matrix stably than other immobilization method^37^,^38^. Moreover, when the cells were immobilized on the PGO (PGO@*P. denitrificans*), the concentration of DMF was dropped off continuously until 0 mg/L by SAB process. In consideration of the advantages mentioned above, PGO@*P. denitrificans* was used in the treatment of relatively high concentration DMF from aqueous solution (2000 mg/L) without any pre-treatment, which made the adsorption-biodegradation process more simple and economical. On the other hand, PGO@*P. denitrificans* also showed an excellent recycle performance, which made the whole process more practical and promising.

## Results and Discussion

### Characterization of GO and PGO

**Figure 2:**
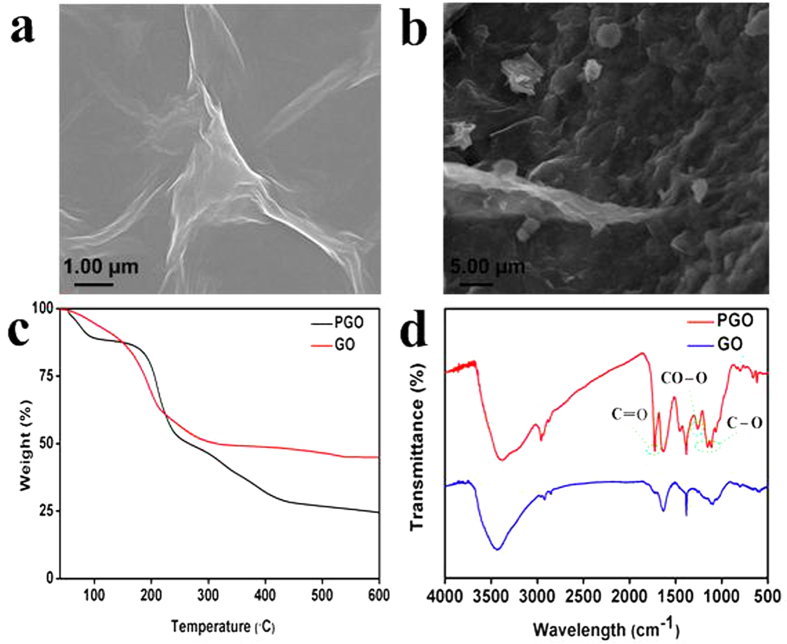
The SEM images of GO (a) and PGO (b), TGA curves of GO and PGO obtained in nitrogen atmosphere (c), FT-IR spectra of GO and PGO (d).

The microstructure and morphology of GO and PGO were analyzed by SEM (Fig. 2). The morphology of GO was shown in Fig. 2a, which presented a laminated structure with smooth surface and wrinkled edge. However, the PGO showed a significantly rougher surface than GO (Fig. 2b), which suggested that the polymers were successfully grafted to the surface of GO.

The amount of polymers that modified on the surface of GO was studied by TGA. Fig. 2c showed the weight loss of the samples. GO was thermally more unstable than PGO and it suffered about 30% weight loss at around 200 °C, which might be owed to the thermal decomposition of the unstable oxygen-containing groups. However, PGO showed a slighter weight loss at around 200 °C compared with GO. In the range between 100 and 600 °C, GO was found to have about 50 wt % weight loss. At the same time, the weight loss of PGO was 75% at 100–600 °C, which resulted from the thermal degradation of polymers and the loss of oxygen-containing functional groups on the GO sheets. So the polymers in PGO were calculated to be approximately 25 wt %.

The FT-IR spectra of GO and PGO were shown in Fig. 2d. Different oxygen-containing functional groups were found in the FT-IR spectrum of GO, the characteristics bands appeared at 3400 cm^−1^ (-OH stretching band), 1730 cm^−1^ (C=O bending of COOH), 1620 cm^−1^ (C-OH vibration frequency), 1380 cm^−1^ (C=C bending) and 1100 cm^−1^ (C-O-O bending). Compared with GO, the obvious increase in the peak at 1730 cm^−1^ could be attributed to the C=O bending of PGO, which also indicated the polymers were grafted to GO successfully. In addition, new bands appeared in the range between 1300–1050 cm^−1^ were the C-O stretching vibration of the polymers in PGO. These changes in FT-IR spectra confirmed the presence of poly(MAA-*co*-BMA) on the surface of GO.

### Adsorption capacity of GO and PGO

**Figure 3:**
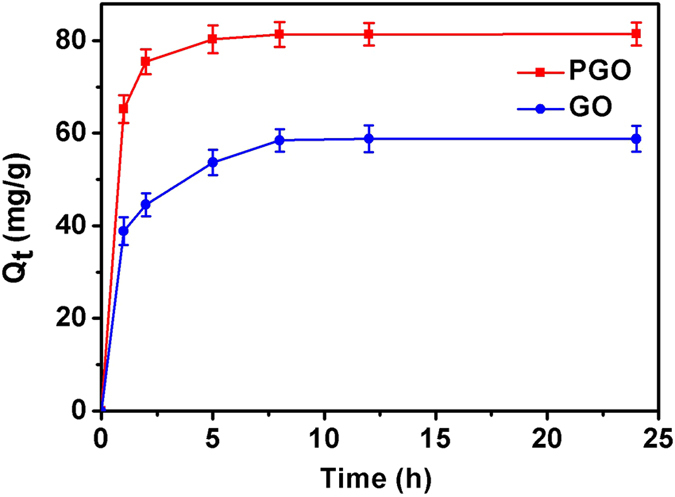
Adsorption capacity of GO and PGO (dry weight of GO = 100 mg, dry weight of PGO = 100 mg, initial DMF concentration = 1000 mg/L, volume of DMF solution = 50 mL and T = 30 °C).

To investigate the adsorption performance of GO and PGO, equilibrium adsorption experiments were carried out in the 250 mL flasks containing 100 mg of diverse adsorbents (GO and PGO) and 50 mL aqueous solution with an initial DMF concentration of 1000 mg/L. And the flasks were shaken in the constant temperature oscillator at 30 °C and 150 r/min. As shown in Fig. 3, for GO and PGO, the adsorption rate were fast at the initial stage and gradually slowed until equilibrium after shaking for about 8 h and 5 h, respectively. For the same initial DMF concentration of 1000 mg/L, the maximal equilibrium adsorption amount of PGO was significantly increased by 40% compared with GO. PGO had more abundant oxygen-containing groups than GO due to polymers on its surface, which formed the Van der Waals force between DMF molecules and polymers, therefore, PGO showed an obviously higher adsorption capacity than GO.

### Acclimatization

**Figure 4:**
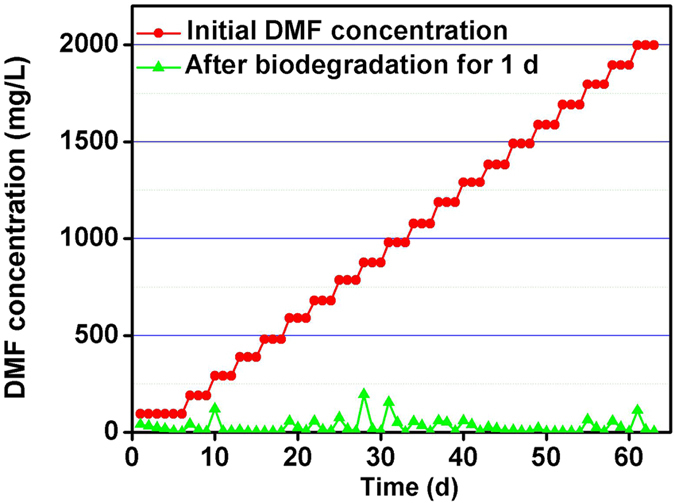
Acclimatization of *P. denitrificans*.

Acclimatization process of *P. denitrificans* cells is shown in Fig. 4. During this process, the DMF concentration was increased 100 mg/L every time when the DMF concentration decreased to 0 mg/L. After acclimatization for 33 days, the *P. denitrificans* could biodegrade DMF with the initial concentration of 1000 mg/L completely without glucose, which indicated that the *P. denitrificans* was capable of utilizing DMF as a sole source of carbon and nitrogen. With the increasing of DMF concentration continually, the *P. denitrificans* could attain a higher biodegradability (2000 mg/L). DMF could be biodegraded under a certain concentration ranged from 0 to 2000 mg/L.

### Biodegradation of DMF by *P. denitrificans*

**Figure 5:**
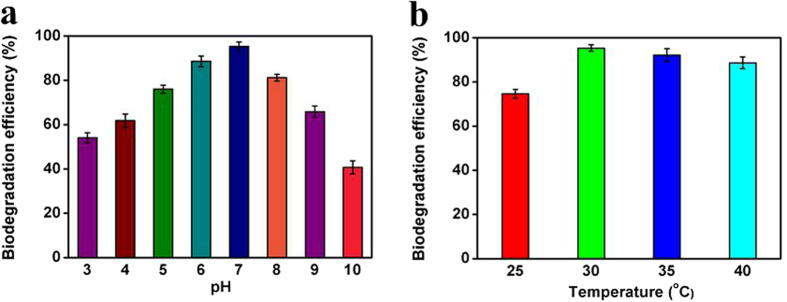
Effect of pH (**a**) and temperature (**b**) on biodegradation efficiency of DMF (initial DMF concentration = 1000 mg/L, volume of DMF solution = 50 mL, wet weight of *P*. denitrificans = 2.0 g).

Before the biodegradation experiments, the optimal conditions of the biodegradation of DMF by *P. denitrificans* cells need to be determined first, and batch experiments were carried out at different pH (3–10) and temperatures (25–40 °C) respectively with the same initial DMF concentration of 1000 mg/L. The results of the optimal conditions for the biodegradation of DMF by *P. denitrificans* cells were shown in Fig. 5, which indicated that it had a best biodegradation rate at the optimized temperature of 30 °C and the optimum pH value near neutrality. Then all the following experiments were carried out under this optimal condition (T = 30 °C, pH = 7).

**Figure 6:**
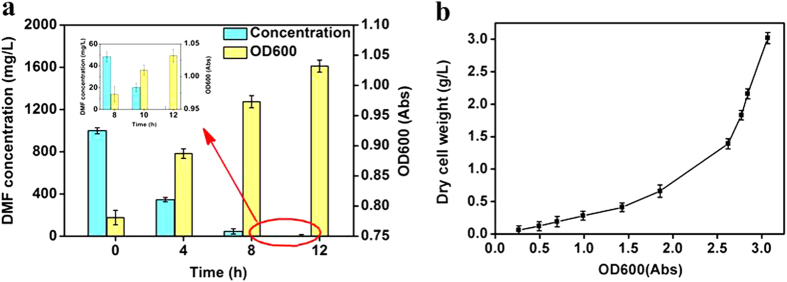
(**a**) Biodegradation curve and OD600 of free P. denitrificans (initial DMF concentration = 1000 mg/L, volume of DMF solution = 50 mL, wet weight of P. denitrificans = 2.0 g, pH = 7 and T = 30 °C) and (**b**) the relative conversion curve of OD600 and DCW.

The biodegradation efficiency of free cells was detected under the optimal conditions. The changes of DMF concentration and OD600 were represented in Fig. 6a. It could be seen that *P. denitrificans* alone was able to degrade DMF completely within 12 h when the initial concentration was 1000 mg/L. And the increase amount of *P. denitrificans* cells was calculated according to the relation curve between OD600 and dry cell weight (DCW) (Fig. 6b) as 6.85 mg.

### Immobilization

**Figure 7:**
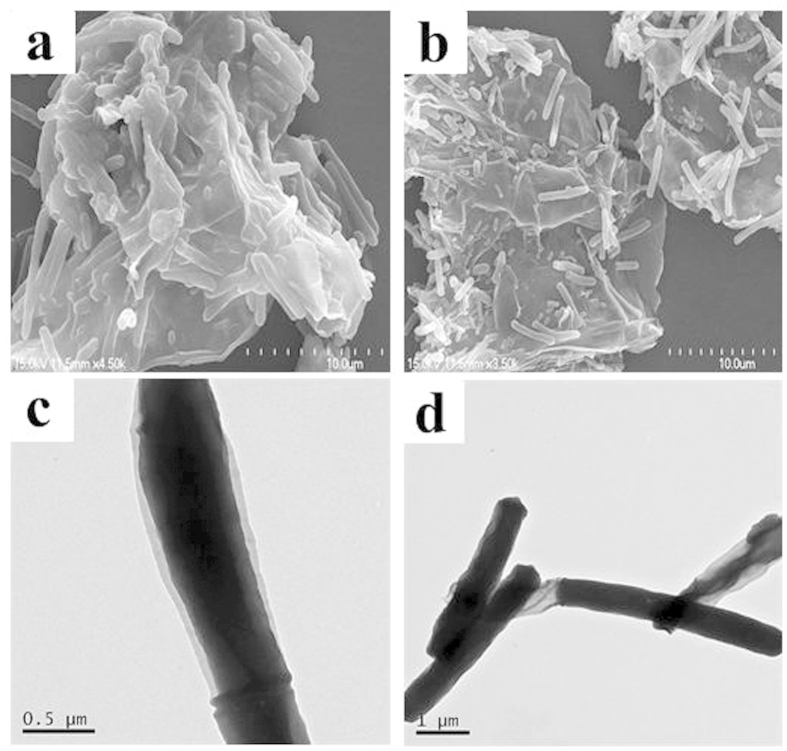
The SEM images (a,b) and TEM images (c,d) of PGO@*P. denitrificans*.

The immobilization process was achieved by covalent coupling. *P. denitrificans* cells had many innate amine groups on the surface, so the bacterial cells could be immobilized onto PGO stably which contained many carboxyl groups (-COOH) on the surface by direct covalent chemical conjugation. N-hydroxy succinimide (NHS) as a robust and highly reactive chemical for introducing functional surface of component to cell membrane was used to accomplish the immobilization process, which could avoid loss of bacterial cells viability. The acclimatized *P. denitrificans* cells were immobilized onto PGO (PGO@*P. denitrificans*) at 30 °C in the constant temperature oscillator (150 r/min). The microstructure and morphology of PGO@*P. denitrificans* were studied by SEM and TEM after freeze-dried at −40 °C for 6 h. As shown in Fig. 7, the acclimatized *P. denitrificans* cells were immobilized onto sheet-shaped PGO firmly by covalent coupling process and also form a large amount of biomass. In addition, it could be clearly seen that the morphology of bacterium cells were well-shaped rodlike structures, which also indicated the PGO had no antimicrobial effect to *P. denitrificans* cells^39^.

Subsequently, the amount of *P. denitrificans* cells immobilized onto PGO was discussed and batch experiments were carried out in the 250 mL flasks at 30 °C. Each flask contained 50 mL PBS solution and the same weight of *P. denitrificans* cells and the amount of bacterium cells was calculated according to the relation curve between OD600 and DCW (Fig. 6b). Then, PGO was added (10 mg, 15 mg and 20 mg) to the flasks to accomplish the immobilization process. As shown in Table 1, for 50 mg (dry cell weight) of *P. denitrificans* cells, the maximum amount of PGO that coupled to the surface of bacterium cells was approximately 15 mg. Then the amount of *P. denitrificans* cells and PGO used for the next adsorption-biodegradation process were followed by this ratio.

**Figure 8:**
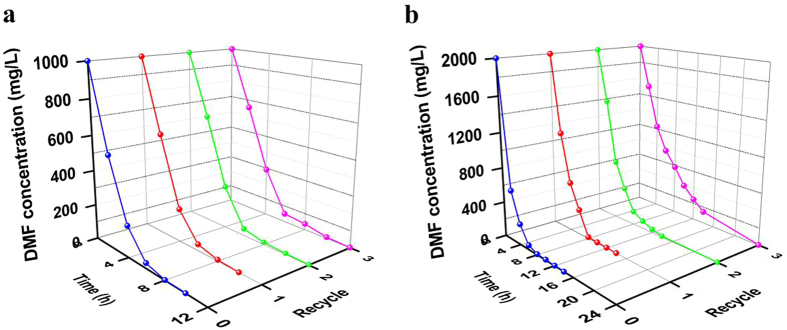
The SAB process of PGO@*P. denitrificans* at different initial DMF concentrations (wet weight of PGO@*P. denitrificans* = 2.0 g, volume of DMF solution = 50 mL, pH = 7 and T = 30 °C).

**Figure 9:**
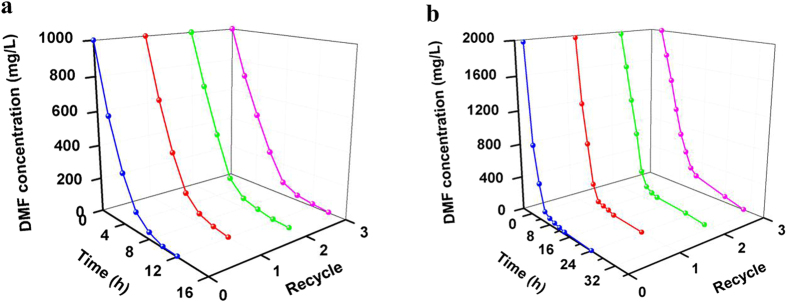
The SAB process of poly(MAA-*co*-BMA)@*P. denitrificans* at different initial DMF concentrations (wet weight of poly(MAA-*co*-BMA)@*P. denitrificans* = 2.0 g, volume of DMF solution = 50 mL, pH = 7 and T = 30 °C).

The PGO@*P. denitrificans* and poly(MAA-*co*-BMA)@*P. denitrificans* were directly used in the removal of high-concentrated DMF from water.The SAB efficiency and recycle performance of PGO@*P. denitrificans* were detected under the optimal conditions and the results were shown in Fig. 8. DMF was removed completely within 10 h from the initial concentration of 1000 mg/L by PGO@*P. denitrificans* (Fig. 8a), which showed a significantly higher efficiency than free cells (Fig. 6a) with the same wet weight of materials even after recycled for 3 times. In addition, the initial DMF concentration of 2000 mg/L could be removed completely just need 14 h by PGO@*P. denitrificans* (Fig. 8b). As can be seen in Fig. 8, the whole process could be divided into two stages. During the first stage within about 4 h, the concentration of DMF decreased quickly, which indicated that PGO played a main role in this adsorption process and most of DMF molecules were adsorbed. Then in the second stage, the adsorption of PGO attained equilibration and the decrease of DMF concentration attributed to the biodegradation of *P. denitrificans*. Besides, the concentration of DMF was dropped off continuously by biodegradation process, which was clearly different from ordinary adsorption process of PGO that maintained at an equilibrium adsorption amount and could not remove DMF from water completely.As can be seen in Fig. 9, DMF was removed completely within 12 h and 24 h for the initial concentration of 1000 mg/L and 2000 mg/L by poly(MAA-*co*-BMA)@*P. denitrificans*, respectively. Thus, PGO@*P. denitrificans* showed a significantly higher SAB efficiency than poly(MAA-*co*-BMA)@*P. denitrificans*, which could be attributed to the relatively large specific area of PGO.

The results of recycle performances of PGO@*P. denitrificans* to the different initial concentrations of DMF were also presented in Fig. 8. After recycling for 3 times, DMF could be removed completely from aqueous solutions within 12 h and 24 h for the initial DMF concentrations of 1000 mg/L and 2000 mg/L, respectively. Although, the processing efficiency of cells-immobilized PGO showed a bit lower than the first process, it still displayed an excellent recycle performance for removal of DMF from aqueous solution. From above we can conclude that PGO@*P. denitrificans* showed not only remarkable removal efficiency but also an excellent recycle performance in the treatment of DMF wastewater compared with free cells and simple material. As can be seen in Fig. 9, after recycled for 3 times, DMF could be removed completely within 14 h and 30 h for the initial DMF concentrations of 1000 mg/L and 2000 mg/L by poly(MAA-*co*-BMA)@*P. denitrificans*, respectively. It is worth noting that poly(MAA-*co*-BMA)@*P. denitrificans* still showed a poorer recycle performance than PGO@*P. denitrificans* obviously.

In order to analyze the SAB kinetic mechanisms, the kinetic data obtained from the batch experiments were investigated with pseudo-first-order and pseudo-second-order kinetic models, respectively.

The pseudo-first-order kinetic model can be expressed by the following equation:


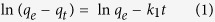


which can be rearranged as:


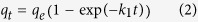


where *q*_t_ is the amount of DMF processed at time t (mg/g); *q*_e_ is the amount of DMF processed at equilibrium (mg/g); and *k*_1_ is the rate constant of pseudo-first-order kinetic model (min^−1^). The *q*_e_ and *k*_1_ values were obtained by plotting *q*_t_ versus *t*.

The pseudo-second-order kinetic model of Ho and McKay can be written as follows:


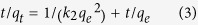


which can be rearranged as:


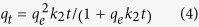


where *k*_2_ is the rate constant of pseudo-second-order kinetic model (g/mg min). The *q*_e_ and *k*_2_ values were calculated by plotting *q*_t_ versus *t*.

The adsorption kinetic parameters were presented in Table 2. For the initial DMF concentration of 1000 mg/L, the correlation coefficient (*R*^2^) in pseudo-second-order model (0.998) was closer to 1.0 than that in pseudo-first-order model (0.959). Similarly, the correlation coefficient (*R*^2^) in pseudo-second-order model (0.996) was also higher than that in pseudo-first-order model (0.944) for the initial DMF concentration of 2000 mg/L. According to the values of *R*^2^, it can be concluded that adsorption kinetics fit better to the pseudo-second-order model than to the pseudo-first-order model.

**Figure 10:**
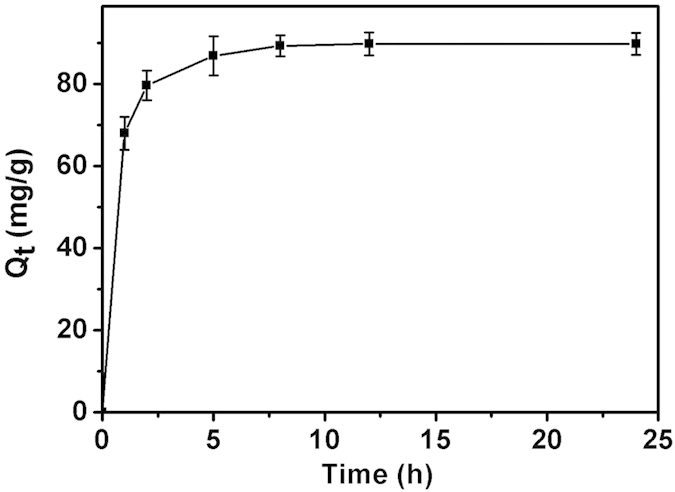
Adsorption capacity of PGO@*P. denitrificans* (dry weight of PGO = 100 mg, initial DMF concentration = 1000 mg/L, volume of DMF solution = 50 mL and T = 30 oC).

The adsorption property of PGO@*P. denitrificans* after freeze-dried was studied subsequently and the result was shown in Fig. 10. As can be seen, for the same initial DMF concentration of 1000 mg/L, the maximal equilibrium adsorption amount of PGO@*P. denitrificans* was increased slightly than PGO (Fig. 3), which could be attributed to a small amount of DMF adsorbed onto the surface of bacterial cells.

## Conclusions

Synthesis of GO with copolymers containing MAA and BMA was achieved to prepare a novel PGO *via* free radical solution polymerization. The PGO was used as an adsorbent as well as a carrier in the treatment of high-concentrated DMF. The PGO@*P. denitrificans* system could cleanse high-concentrated DMF (2000 mg/L) wastewater completely, which showed not only remarkable removal efficiency but also an excellent recycle performance compared with free cells and simple material. Moreover, PGO@*P. denitrificans* needed no pretreatment or regeneration procedure during the recycle process, which made the whole process more simple and practical. In summary, the two-step method based on SAB has a great prospect in the treatment process of wastewater containing high-concentrated DMF.

## Methods

### Materials

Tryptone and yeast extract were purchased from Suzhou Biogene Biotechnology Co.,Ltd. Graphite powder, sulfuric acid (H_2_SO_4_), hydrochloric acid (HCl), hydrogen peroxide (H_2_O_2_), dimethylacetamide (DMAC), triethylamine (TEA), methacryloyl chloride (MAC), azobisisobutyronitrile (AIBN), methacrylic acid (MAA), butyl methacrylate (BMA), N-hydroxy succinimide (NHS), N,N’-Dicyclohexylcarbodiimide (DCC), 4-dimethylaminopyridine (DMAP), cyclohexanone, petroleum ether, ether and inorganic salts were all in analytical reagent grade were commercially obtained from Sinopharm Chemical Reagent Co.,Ltd. and used as received without further treatment. HPLC grade methanol was purchased from Shanghai Qiangshun Chemical Reagent Co.,Ltd. HPLC grade DMF was obtained from Tedia Company Inc.

### Bacterial culture and acclimatization

Before experiments, Luria Bertani (LB) liquid medium (Tryptone, 10 g/L; Yeast extract, 5 g/L; NaCl, 10 g/L), mineral salt medium (MM1) solution (K_2_HPO_4_, 6.3 g/L; KH_2_PO_4_, 1.8 g/L; MgSO_4_·7H_2_O, 0.1 g/L; MnSO_4_·4H_2_O, 0.1 g/L; CaCl_2_·2H_2_O, 0.1 g/L; FeSO_4_·7H_2_O, 0.1 g/L; NaMoO_7_·7H_2_O, 0.006 g/L), Phosphate Buffered Saline (PBS) solution and all instruments were autoclaved at 120 °C for 20 min, and the DMF and glucose solution were separately sterilized by 0.22 μm membrane filter before use.

A pure strain of *P. denitrificans* (ATCC 19367) was purchased from Shanghai Fuxiang Biotechnology Co.,Ltd. Firstly, the bacterium was inoculated in the LB liquid medium, and incubated at 30 °C and 150 r/min on the constant temperature oscillator (SHA-CA) until it grew into the logarithmic phase. The *P. denitrificans* was harvested by centrifuging at 6000 rpm for 10 min and then transferred in a 50 mL MM1 solution with 1000 mg/L of glucose (usually used as the source of carbon) and incubated at 30 °C, 150 r/min.

The acclimatization process was accomplished by increasing the DMF concentration (100 mg/L every time) gradually and in the meanwhile decreasing the glucose concentration (100 mg/L every time) gradually in MM1 solution until 0 mg/L. During this time, the strain was capable of utilizing DMF as a sole source of carbon and nitrogen. Constantly increased the DMF concentration up to 2000 mg/L and completed the acclimatization process. The acclimatized bacteria were used for next DMF biodegradation and immobilization experiments.

### Synthesis of GO

GO was prepared from graphite powder by a modified Hummers method reported in the previous study^40^,^41^. Briefly, graphite (3.0 g) and NaNO_3_ (1.5 g) were mixed in concentrated H_2_SO_4_ (69 mL), and the mixture was cooled using an ice bath to 0 °C. Then, KMnO_4_ (9.0 g) was added to the suspension slowly to keep the reaction temperature lower than 20 °C. The reaction system was warmed to 35 °C and vigorously stirred for 7 h. Next, additional KMnO_4_ (9.0 g) was added in one portion, and the reaction was still stirred at 35 °C for 12 h. After that, the reaction mixture was cooled to room temperature and poured into ice water (400 mL), then 30% H_2_O_2_ (3 mL) was added dropwise to reduce the residual KMnO_4_ until no bubbles appeared, and the color of solution from brown turned to yellow. Then the mixture was centrifuged and washed with HCl (5%) to remove metal ions and rinsed with deionized water repeatedly to remove acid. The product was dispersed in deionized water to make a GO aqueous dispersion and then purified by dialysis for two weeks to remove the salt impurities and remaining acid. Subsequently, the solution was centrifuged at 11000 rpm for 20 min to remove redundant impurities. Finally, the supernatant liquor was obtained as GO solution and then was dried in vacuum desiccator. The dried GO was used for the next experiments.

### Synthesis of poly(MAA-*co*-BMA)

Poly(MAA-*co*-BMA) was prepared *via* free radical solution polymerization with MAA and BMA as monomers and AIBN as the initiator. The monomers mixture (MAA, 70 μL; BMA, 385 μL) and initiator (AIBN, 8 mg) were added into a 50 mL round-bottomed flask containing 10 mL cyclohexanone. Then, the reaction system was heated to 70 °C and stirred for 8 h under N_2_ atmospheric. Subsequently, the mixture was cooled to room temperature and precipitated in petroleum ether, and then was filtered, washed completely with petroleum ether to remove the residual monomer and dried in vacuum desiccator to obtain poly(MAA-*co*-BMA).

### Synthesis of PGO

PGO was prepared *via* free radical solution polymerization with MAA and BMA as monomers and AIBN as the initiator. Firstly, dried GO (100 mg) and TEA (268 μL) were dispersed in DMAC (40 mL) in a 100 mL round-bottomed flask equipped with a mechanical stirrer and constant pressure funnel (50 mL) that contained MAC (188 μL) and DMAC (20 mL). The TEA (TEA : MAC = 1: 1 in stoichiometric molar ratio) was used for neutralizing the HCl produced from the reaction. The mixture in the constant pressure funnel was added dropwise into the flask under N_2_ atmospheric conditions at 0 °C for 0.5 h. Then the reaction system was warm to room temperature and stirred for 24 h. Subsequently, the product was centrifuged, washed with acetone and dried to give 140 mg solid. Next, the resulting solid was dispersed in 20 mL DMF in a 50 mL round-bottomed flask equipped with a mechanical stirrer. After adding the monomers mixture (MAA, 70 μL; BMA, 385 μL) and the initiator (AIBN, 8 mg) into the flask, the reaction system was heated to 70 °C for 8 h under N_2_ atmospheric conditions. After the system was cooled to room temperature, the mixture was precipitated in ether, and then was filtered, washed completely with ether to remove the residual monomer and dried in vacuum desiccator to obtain PGO.

### Immobilization of *P. denitrificans* cells onto PGO and poly(MAA-co-BMA)

Firstly, 50 mL of acclimatized bacterial cells were incubated at 30 °C in the LB liquid medium, then the free cells were centrifuged and washed with PBS solution for 3 times. Subsequently, dried PGO (100 mg) and NHS (100 mg) were disperse in DMF (40 mL) in a 100 mL round-bottomed flask. Then DCC (300 mg) and DMAP (DCC : DMAP = 1 : 1 in stoichiometric molar ratio) which were used as catalyst were added to the flask and stirred for 24 h at room temperature. Then the mixture was centrifuged and washed with PBS solution for 3 times and added to the bacteria suspension containing 50 mL PBS solution in 250 mL Erlenmeyer flasks. The immobilization process was carried out in the constant temperature oscillator at 30 °C and 150 r/min for about 24 h. The immobilization process of *P. denitrificans* cells onto poly(MAA-*co*-BMA) was consistent with that onto PGO. The PGO@*P. denitrificans* and poly(MAA-*co*-BMA)@*P. denitrificans* were used for next DMF adsorption-biodegradation studies.The amount of PGO coupled to the surface of *P. denitrificans* was estimated by determining the difference of dried PGO weight before and after immobilization.

### Batch experiments

All batch experiments were carried out to investigate the DMF adsorption performance in 250 mL Erlenmeyer flasks shaking in the constant temperature oscillator (SHA-CA).

The adsorption amount of DMF was calculated using the following equation:


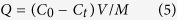


where *Q* (mg/g) represents the adsorption amount of adsorbents, *C*_0_ (mg/L) is the initial DMF concentration, *C*_t_ (mg/L) is the residual DMF concentration at time t, *V* (L) denotes the volume of the solution, and *M* (dry weight, g) is the dosage of adsorbents.

### Analytical methods

The concentration of DMF was determined against a calibration curve of standard DMF solutions of known concentration by high performance liquid chromatograph (HPLC, Agilent 1260 Infinity) using a Accurasil C18 column (4.6 mm × 150 mm inner diameter, 5 mm particle size) at 25 °C, G1311C pumps and Ultraviolet detector (1260 MWD) at 205 nm. The mobile phase consisted of 15 : 85 (v/v) methanol : water at a flow rate of 1 mL/min. And the samples were centrifuged and filtered through 0.22 μm membrane filters before injection. Cell growth was monitored by measuring the optical density (OD) in a UV-Vis spectrophotometer (TU-1901) at the wavelength of 600 nm. And dry cell mass could be known from the OD600 value by using a relation curve which was described by the known quantities. The pH value of solution was determined by a HANNA pH meter. Scanning electron microscopy (SEM) images were obtained on a Hitachi S-4800 electron microscope. Transmission electron microscopy (TEM) images were taken on a Hitachi H600 electron microscope. Fourier transform infrared spectroscopy (FT-IR) was performed on a Nicolet 4700 spectrometer (Thermo Fisher Scientific) in the range of 4000–500 cm^−1^ in KBr pellet at room temperature. Thermogravimetric analysis (TGA) was carried out using a TG209 F1 Libra thermal analyzer from room temperature to 600 °C with heating rate of 10 °C/min and a N_2_ flow rate of 200 mL/min.

## Additional Information

**How to cite this article**: Zheng, Y. *et al.* Efficient simultaneous adsorption-biodegradation of high-concentrated N,N-dimethylformamide from water by *Paracoccus denitrificans*-graphene oxide microcomposites. *Sci. Rep.* **6**, 20003; doi: 10.1038/srep20003 (2016).

## Figures and Tables

**Figure 1:**
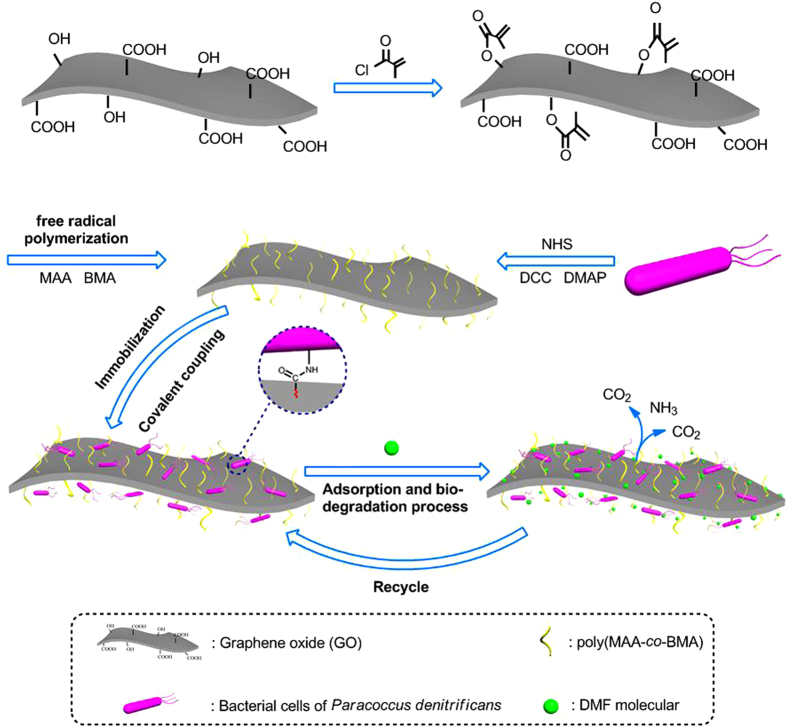
Illustration of PGO synthetic procedure and adsorption-biodegradation process of cells-immobilized PGO for DMF.

**Table 1:** Quantitative analysis of PGO@*P. denitrificans.*

	*Paracoccus denitrificans*(mg)	PGO (mg)	Residual PGO (mg)
A	50	10	0
B	50	15	0
C	50	20	4.5

SAB process and recycle performance of PGO@*P. denitrificans* and poly(MAA-*co*-BMA)@*P. denitrificans*

**Table 2:** Kinetics parameters of pseudo-first-order and pseudo-second-order model for SAB of DMF with different concentrations based on PGO@*P. denitrificans.*

	Pseudo-first-order [*q*_t_ = *q*_e_ (1−exp (−*k*_1_*t*)]			Pseudo-second-order [*t/q*_t_ = 1/(*k*_2_*q*_e_^2^) + *t/q*_e_]		
*C*_0_(mg/L)	*q*_e_ (mg/g)	*k*_1_ (min^−1^)	*R*^2^	*q*_e_ (mg/g)	*k*_2_ (min^−1^)	*R*^2^
1000	17.838	0.0056	0.959	22.645	0.0158	0.998
2000	33.268	0.0099	0.944	35.638	0.0011	0.996
